# 雌激素在非小细胞肺癌中的研究进展

**DOI:** 10.3779/j.issn.1009-3419.2017.07.09

**Published:** 2017-07-20

**Authors:** 小胜 丁, 传昊 汤, 志杰 王, 军 梁

**Affiliations:** 102206 北京，北京大学国际医院肿瘤内科 Department of Oncology, Peking University International Hospital, Beijing 102206, China

**Keywords:** 肺肿瘤, 雌激素, 雌激素受体, 抗雌激素治疗, 表皮生长因子受体酪氨酸激酶抑制剂, Lung neoplasms, Estrogen, Estrogen receptors, Antiestrogens, Epidermal growth factor receptor tyrosine kinase inhibitor

## Abstract

肺癌是目前发病率及死亡率最高的恶性肿瘤之一，其中约85%为非小细胞肺癌（non-small cell lung cancer, NSCLC）。尽管其治疗手段不断提高，但总体预后不容乐观。既往研究已证实雌激素系统参与了NSCLC的发生、发展。越来越多的证据表明，抗雌激素治疗不仅可以逆转NSCLC患者对铂类化疗药物的耐药性，还可以增加人类表皮生长因子受体酪氨酸激酶抑制剂的疗效。本文就雌激素系统及抗雌激素治疗在NSCLC中的作用作一综述。

## Abstract (en)

Lung cancer, of which approximately 85% are non-small cell lung cancer (NSCLC), is one of the most prevalent cancers and the most leading cause of cancer mortality. Despite recent improvements in its treatment, the prognosis remains dismal. Previous studies have clearly proved that estrogen and estrogen receptors (ER) are involve in the pathogenesis and development of lung cancer. More and more evidences showed antiestrogen therapy may reverse the drug-resistance of platinum based chemotherapy in NSCLC patients and can enhance curative effect of epidermal growth factor receptor tyrosine kinase inhibitor. We will review recent progress in the function of estrogen in NSCLC and the treatment based on the ER signaling pathways for NSCLC in this article.

肺癌居全球癌症发病率及病死率首位，其中非小细胞肺癌（non-small cell lung cancer, NSCLC）占肺癌发病率80%以上，虽以手术、放化疗、靶向治疗等为基础的综合治疗使NSCLC治疗效果取得了长足的进步，但生存时间的延长并不满意。既往大量研究证实雌激素可以促进肺癌细胞增殖，抗雌激素治疗可以抑制肿瘤发展，因此有学者提出肺癌也是激素依赖性肿瘤^[1]^。本文就雌激素系统在NSCLC发生、发展中的作用及抗雌激素治疗的相关研究进展作一综述。

## 1 雌激素与NSCLC

### 1.1 雌激素生理功能

雌激素是一种甾体类固醇性激素，主要包括雌二醇（E2）、雌三醇和雌酮，在体内发挥促性器官发育、维持女性正常生理周期及促进骨钙沉积等功能。育龄期女性体内的雌激素主要由卵巢和胎盘产生，而绝经后女性及男性体内的雌激素主要是由内源性雄激素通过芳香化酶通路转化而来。雌激素可以通过基因组途径和非基因组途径两种方式发挥生物学效应，前者由雌激素反应元件介导的经典途径和以AP1为代表的转录因子介导的非经典途径组成，后者则为雌激素与胞膜性雌激素受体（membrane estrogen receptor, mER）结合后通过细胞内信号转导分子的快速激活发挥作用。

### 1.2 雌激素在NSCLC中的表达

在NSCLC组织和肺癌细胞株中运用质谱法均检测出高水平的β雌二醇，癌组织中β雌二醇浓度明显高于非肿瘤组织^[2]^。女性NSCLC患者血清中雌激素水平较同龄正常女性更高，且这种高雌激素水平在治疗后出现显著下降。肺癌患者体内的高雌激素水平及与治疗的关系也为不断发现的证据证实。

### 1.3 雌激素与NSCLC发生、发展和预后

吸烟是肺癌的明确诱因，在吸烟暴露量相同时，女性罹患肺癌的风险要比男性高出2倍以上^[3]^。雌激素具有致癌及促癌作用。E2对肺癌细胞和正常肺成纤维细胞均有促增殖效应，使用E2培育肺癌细胞系可使其增殖能力提高16倍，而正常肺成纤维细胞仅提高2.8倍。在肺癌小鼠移植瘤模型中，E2可显著刺激移植瘤的生长^[4]^。雌激素不仅可以诱导多种原癌基因的活化，还可抑制抑癌基因（如*P53*、*Rb*）的激活来诱发正常细胞恶变^[5]^。多项大样本研究均证实激素替代治疗（hormone replacement therapy, HRT）与肺癌发生风险及预后有关。绝经期女性为了延缓及阻止与绝经相关的疾病常常接受HRT，但临床研究发现长期接受HRT的女性，肺癌的发病风险及死亡风险均明显升高^[6, 7]^。有研究报道接受HRT的女性确诊肺癌时年龄更小（63岁 *vs* 68岁，*P*＜0.000, 1）、生存期更短（39个月 *vs* 79个月，RR=1.97）。妇女健康倡议以16, 608例绝经后女性为研究对象，进行随机对照双盲临床试验，经过5年多的密切随访证实接受HRT的实验组肺癌相关死亡率明显高于对照组，且肿瘤呈现出分化更差、更易转移的趋势^[7]^。研究人员在瑞士选取6, 655例乳腺癌患者，其中3, 066例接受了抗雌激素治疗，结果显示接受抗雌激素治疗的乳腺癌患者尽管其肺癌发病率没有明显下降，但因肺癌导致的死亡率下降了87%^[8]^。但也有研究^[9]^认为绝经后女性接受HRT可降低NSCLC的发病风险。分析上述研究结果差异的原因可能与接受HRT的持续时间有关，短期使用可能具有保护作用，长时间使用则有害。

肿瘤血管内皮生长因子基因的非编码区与雌激素反应元件至少存在60%的同源性，因此雌激素还可以通过增加肿瘤血管内皮生长因子表达来促进肿瘤血管生成^[10, 11]^。雌激素在癌细胞浸润、转移过程中同样扮演着重要角色，其机制可能是通过产生尿激酶型纤维蛋白溶酶原激活物降解细胞外基质、上调Osteopontin和MMP2表达水平等方式促进癌细胞向周围组织浸润和转移^[12-14]^。

Moore等^[15]^分析了绝经前后女性肺癌患者的生存情况，发现绝经前女性与同龄组男性相比并无生存优势，而绝经后女性明显优于同龄组男性。在不吸烟的女性肺腺癌人群中，与绝经后患者相比，未绝经者肿瘤侵袭性更强，生存时间更短^[12]^。

## 2 ER与NSCLC

### 2.1 ER的构成

ER包括2类，一是经典的核受体ERα、ERβ，结合配体后进入细胞核，结合至靶基因上的雌激素反应元件发挥“基因型”调节效应；二是膜性受体，包括经典核受体的膜性成分和属于G蛋白偶联受体家族的GPER1，其通过快速的非基因组途径发挥作用。目前发现ERα存在3种亚型，即ERα66、ERα46、ERα36，ERβ存在5种亚型，即ERβ1-5。ERα主要分布于卵巢、乳腺和子宫内膜，而ERβ多表达于肺和卵巢。

### 2.2 ER在NSCLC中的表达

自从Chaudhuri首次报道肺癌组织表达ER，此后研究人员分别采用IHC、Western blot及RT-PCR等多种方法对肺癌组织中ER的表达及功能展开了研究。ERα和ERβ在NSCLC中的表达水平文献报道差异较大，前者在0-96.8%之间，后者在9%-98%之间。GPER1在肺癌组织中表达率高于70%，而在癌旁组织及正常肺组织中表达率却低于3%。ERα和ERβ均可表达于NSCLC细胞质和细胞核中^[16-19]^，而且ER表达水平与肿瘤组织学类型和分化程度等临床特征有关，NSCLC高于小细胞肺癌，腺癌高于鳞癌^[17]^，这也是女性腺癌发病率高于男性的原因之一。通过检测不同分化程度的肺癌组织，发现ER表达与肺癌细胞分化更佳相关^[20]^。

### 2.3 ER与NSCLC的预后及治疗

#### 2.3.1 ER与NSCLC预后

基础及临床研究均证实ER的表达水平对预测NSCLC患者预后有重要的意义。Kawai等^[16]^发现ERα及ERβ均广泛表达于NSCLC组织中，ERα(-)/ERβ(+)的患者预后明显优于其他亚组。Rades等^[21]^分析了接受放疗的Ⅱ期/Ⅲ期NSCLC患者，发现ERα表达的患者存在更低的局部控制率和3年生存率（10% *vs* 54%, *P*＜0.05）。其他研究^[22, 23]^也进一步证实ERα表达是NSCLC不良的预后因素。NSCLC中ERβ特异性激动剂可激活其下游信号分子发挥促进肿瘤生长作用，而ERα不能，越来越多的研究证实ERβ是雌激素在肺部发挥作用的主要功能受体^[24, 25]^，其在NSCLC中的预后意义一直备受关注。Chen等^[26]^报道在187例接受手术治疗的Ⅱ期/Ⅲ期NSCLC患者中，伴有ERβ高表达的患者中位生存期更长，Skov等^[27]^也发现表达ERβ的男性NSCLC患者死亡率更低。但也有研究与之不一致，有研究^[28]^报道存在ERβ1细胞核染色的晚期肺癌患者生存时间明显缩短，Skjefstad等^[29]^在女性肺癌中也发现这种情况，Stabile等^[30]^则发现细胞浆中高表达ERβ的患者预后更差（1.87年 *vs* 3.5年，*P*=0.021）。有文献^[31]^报道ERβ在细胞质和细胞核中的作用存在差异。因此，NSCLC中ERβ预后意义的差异，可能与ERβ1-5的表达、分布及功能不同有关^[32]^。

#### 2.3.2 ER与表皮生长因子受体酪氨酸激酶抑制剂（epidermal growth factor receptor tyrosine kinase inhibitor, EGFR-TKIs）治疗和抗雌激素治疗

目前人类EGFR-TKIs已广泛应用于晚期肺癌的治疗，在取得显著疗效的同时明显改善了患者的生活质量。近年来多位学者提出在肺癌、乳腺癌及头颈部肿瘤等多种实体瘤中，ER与EGFR下游通路之间存在信号交叉。有研究^[33]^证实ER和EGFR信号通路可以协同激活P42/P44 MAPK通路，使用siRNA干扰ERα和ERβ表达，可以显著减弱NSCLC细胞的体外增殖。Tang等^[4, 34]^报道雌激素可以激活MAPK和Akt分子信号通路来促进小鼠肺腺癌的生长。Pinton等^[35]^在研究恶性胸膜间皮瘤ER和EGFR相互作用时发现若过表达ERβ，则癌细胞对Gefitinib耐药，而当沉默ERβ表达时，EGFR呈现磷酸化且细胞恢复对Gefitinib的敏感。临床研究中，Nose等^[36-38]^报道存在ERβ核表达的患者有更高的*EGFR*突变率及更长的EGFR-TKIs无进展生存期（progression-free survival, PFS）。我们前期的研究也发现进展期NSCLC组织存在ERβ细胞浆和细胞核表达，而且存在胞浆/胞核共表达的患者使用EGFR-TKIs时mPFS明显更短（3.0个月 *vs* 7.8个月，*P*＜0.05），原因可能与肺癌细胞中存在多种ERβ亚型以及ERβ非基因组途径快速激活导致EGFR下游信号分子活化有关^[39]^。基于ER和EGFR分子通路之间存在的信号交叉，有学者提出抗雌激素治疗和EGFR-TKIs治疗之间可能存在协同抗癌效应^[33, 39-41]^。在肺癌细胞株A549和H1650中，三苯氧胺和吉非替尼联合用药比任一单药都可以更加显著地增加癌细胞的凋亡和降低细胞增殖^[40]^，还有研究^[42, 43]^证实两类药物联合使用对抑制肺癌细胞生长效果更好。动物实验也证实与氟维司群或特罗凯单药相比，联合用药可以更有效地抑制肿瘤的生长^[33, 43]^。我们的研究也发现肺癌细胞株对吉非替尼耐药后可被ER拮抗剂-氟维司群逆转^[39]^。我们在临床工作中遇到1例57岁伴组织*EGFR* 19外显子敏感突变的女性Ⅳ期肺腺癌患者，在接受吉非替尼治疗8.7个月后肺部原发病灶进展，加用局部放疗后肿瘤仍快速进展，并出现胸腔积液和骨转移，免疫组化证实部分腺癌细胞同时在细胞浆和细胞核中表达ERβ，在充分知情同意的情况下加用氟维司群1.5个月后患者胸腔积液明显减少，肺部病灶缩小，使PFS延长了3个月^[39]^。氟维司群联合吉非替尼的临床研究显示出良好的安全性，对女性晚期NSCLC尤其是高表达ERβ的患者，可增加生存时间。Ⅱ期临床试验显示在EGFR阳性肺癌患者中，联合用药较单独用药可延长患者生存期^[44]^。目前已有多项临床研究正在进行中，以进一步评估联合氟维司群和EGFR抑制剂对晚期NSCLC的临床价值。

#### 2.3.3 ER与其他信号通路的活化

NSCLC细胞中ER和IGFR信号通路之间也存在信号交叉，雌激素可通过活化ERβ促进IGFR表达上调来促进肿瘤进展^[45, 46]^。近期有研究^[47]^报道雌激素还可促进FGF2释放、活化肺癌细胞中FGFR信号通路进而促进癌细胞增殖，氟维司群联合FGFR抑制剂AZD4547明显降低了肿瘤的生长、Ki67指数及干细胞标志物的表达。

#### 2.3.4 ER与肺癌化疗

一项早期临床试验显示三苯氧胺辅助治疗肺癌可以取得良好疗效，尤其对ER阳性患者的有效率可达58.3%。另一项研究中，三苯氧胺与顺铂联合治疗NSCLC同样显示较好的疗效及耐受性。Che^[48]^使用高剂量三苯氧胺（每天单剂量150 mg/m^2^）联合顺铂化疗，增加了顺铂化疗的敏感性。近期研究发现氟维司群还可降低肿瘤细胞的间充质特性进而增加免疫治疗及化疗的敏感性^[49]^。此外，雌激素还可通过ER导致肺癌患者顺铂化疗耐药，原因可能与ERα/ERβ相对表达水平变化及P53通路活化水平有关^[50]^。

## 3 芳香化酶与NSCLC

### 3.1 芳香化酶的结构和生理功能

芳香化酶是微粒体细胞色素P450酶超家族的成员，是P450产物中唯一由单基因编码的复合酶，由位于染色体15q21.2区位的CYP19基因编码。芳香化酶可氧化脱去C19类固醇（雄烯二酮和睾酮）的19-甲基，使A环芳香化，从而转变成C18雌激素（雌酮和β雌二醇），是雌激素合成的最后一步限速酶。

### 3.2 芳香化酶在NSCLC中的表达

芳香化酶在肺癌组织和肺癌细胞系中均存在表达^[2, 51, 52]^，促进癌组织中β雌二醇的自身合成。有研究^[6]^通过免疫组织化学发现85%NSCLC组织中表达芳香化酶，且多伴随ERβ的表达。还有研究^[7]^发现肺癌组织中芳香化酶表达与患者雌激素水平呈正相关，而在对照的正常组织中未检测到芳香化酶表达。

### 3.3 芳香化酶与NSCLC预后及治疗

#### 3.3.1 芳香化酶与NSCLC预后

芳香化酶水平与预后的关系在乳腺癌等激素依赖性肿瘤中已被广泛证明，而在NSCLC中的报道却寥寥无几。Mah等^[53]^发现在早期不吸烟的老年NSCLC女性中，芳香化酶高表达患者预后明显更差，可作为预测预后的一种生物标记物。Mikihiro和Kazumi也发现高表达芳香化酶的NSCLC患者术后无复发生存时间和总生存期更短。

#### 3.3.2 芳香化酶与NSCLC治疗

芳香化酶促进肿瘤组织局部产生高浓度的雌激素进而促进肿瘤细胞增殖、抑制凋亡。对于绝经后女性而言，体内的循环雌激素水平较低，肿瘤组织表达芳香化酶产生的雌激素可能是患者雌激素水平高低的决定因素。芳香化酶抑制剂可与芳香化酶结合并抑制其活性，阻断内源性雄激素向雌激素的转化，从而下调局部雌激素水平，抑制肺癌的进展。多项基础研究均发现在肺癌细胞系中使用芳香化酶抑制剂可明显抑制癌细胞的增殖^[51, 52]^，动物实验也发现阿那曲唑可显著降低NSCLC异种移植瘤的生长。在NSCLC患者化疗过程中，芳香化酶抑制剂联合顺铂具有明显的协同抗癌效应，可以更加强烈地抑制肺癌的进展，降低肿瘤侵袭性。以芳香化酶为靶标的研究一直都是抑制剂研究的热点，其为肺癌的治疗提供了一种新的选择。

## 4 展望

雌激素系统与激素依赖性肿瘤的研究由来已久，而与肺癌关系的探索才刚刚起步，越来越多的研究证实雌激素系统在肺癌发生、发展及预后方面起着重要作用，抗雌激素治疗在与化疗、靶向药物联合治疗NSCLC方面也取得了一定的成绩。笔者相信肺癌抗雌激素治疗是值得探索的治疗方式，深入研究其中的分子机制对NSCLC的预防、治疗有着重要的临床意义。

## References

[1] Beattie CW, Hansen NW, Thomas PA (1985). Steroid receptors in human lung cancer. Cancer Res.

[2] Niikawa H, Suzuki T, Miki Y (2008). Intratumoral estrogens and estrogen receptors in human non-small cell lung carcinoma. Clin Cancer Res.

[3] Papadopoulos A, Guida F, Leffondre K (2014). Heavy smoking and lung cancer: are women at higher risk? Result of the ICARE study. Br J Cancer.

[4] Tang H, Liao Y, Chen G (2013). Estrogen and insulin-like growth factor 1 synergistically promote the development of lung adenocarcinoma in mice. Int J Cancer.

[5] Liu W, Konduri SD, Bansal S (2006). Estrogen receptor-alpha binds p53 tumor suppressor protein directly and represses its function. J Biol Chem.

[6] Ganti AK, Sahmoun AE, Panwalkar AW (2006). Hormone replacement therapy is associated with decreased survival in women with lung cancer. J Clin Oncol.

[7] Chlebowski RT, Schwartz HG, Wakelee H (2009). Oestrogen plus progestin and lung cancer in postmenopausal women (Women's Health Initiative trial): a posthoc analysis of a randomised controlled trial. Lancet.

[8] Bouchardy C, Benhamou S, Schaffar R (2011). Lung cancer mortality risk among breast cancer patients treated with anti-estrogens. Cancer.

[9] Schwartz AG, Wenzlaff AS, Prysak GM (2007). Reproductive factors, hormone use, estrogen receptor expression and risk of non-small-cell lung cancer in women. J Clin Oncol.

[10] Applanat MP, Buteau-Lozano H, Herve MA (2008). Vascular endothelial growth factorisa target gene for estrogen receptor and contributes to breast cancer progression. Adv Exp Med Biol.

[11] Hyder SM, Nawaz Z, Chiappetta C (2000). Identification of functional estrogen response elements in the gene coding for the potent angiogenic factor vascular endothelial growth factor. Cancer Res.

[12] Hsu LH, Liu KJ, Tsai MF (2015). Estrogen adversely affects the prognosis of patients with lung adenocarcinoma. Cancer Sci.

[13] Fan S, Liao Y, Liu C (2017). Estrogen promotes tumor metastasis via estrogen receptor beta-mediated regulation of matrix-metalloproteinase-2 in non-small cell lung cancer. Oncotarget.

[14] Rodriguez-Lara V, Ignacio GS, Cerbón Cervantes MA (2017). Estrogen induces CXCR4 overexpression and CXCR4/CXL12 pathway activation in lung adenocarcinoma cells *in vitro*. Endocr Res.

[15] Moore KA, Mery CM, Jaklitsch MT (2003). Menopausal effects on presentation, treatment, and survival of women with non-small cell lung cancer. Ann Thorac Surg.

[16] Kawai H, Ishii A, Washiya K (2005). Estrogen receptor alpha and beta are prognostic factors in non-small cell lung cancer. Clin Cancer Res.

[17] Mollerup S, Jorgensen K, Berge G (2002). Expression of estrogen receptors alpha and beta in human lung tissue and cell lines. Lung Cancer.

[18] Kawaguchi T, Koh Y, Ando M (2016). Prospective analysis of oncogenic driver mutations and environmental factors: Japan Molecular Epidemiology for Lung Cancer Study. J Clin Oncol.

[19] Deng F, Li M, Shan WL (2017). Correlation between epidermal growth factor receptor mutations and the expression of estrogen receptor-β in advanced non-small cell lung cancer. Oncol Lett.

[20] Chen XQ, Zheng LX, Li ZY (2017). Clinicopathological significance of oestrogen receptor expression in non-small cell lung cancer. J Int Med Res.

[21] Rades D, Setter C, Dahl O (2012). The prognostic impact of tumor cell expression of estrogen receptor-alpha, progesterone receptor, and androgen receptor in patients irradiated for non-small cell lung cancer. Cancer.

[22] Raso MG, Behrens C, Herynk MH (2009). Immunohistochemical expression of estrogen and progesterone receptors identifies a subset of NSCLCs and correlates with *EGFR* mutation. Clin Cancer Res.

[23] Olivo-Marstone SE, Mechanic LE, Mollerup S (2010). Serum estrogen and tumor-positive estrogen receptor-alpha are strong prognostic classifiers of non-small-cell lung cancer survival in both men and women. Carcinogenesis.

[24] Zhang G, Yanamala N, Lathrop KL (2010). Ligand-independent antiapoptotic function of estrogen receptor-beta in lung cancer cells. Mol Endocrinol.

[25] Márquez-Garbán DC, Chen HW, Fishbein MC (2007). Estrogen receptor signaling pathways in human non-small cell lung cancer. Steroids.

[26] Chen XQ, Zheng LX, Li ZY (2017). Clinicopathological significance of oestrogen receptor expression in non-small cell lung cancer. J Int Med Res.

[27] Skov BG, Fischer BM, Pappot H (2008). Oestrogen receptor beta over-expressionin males with non-small cell lung cancer is associated with better survival. Lung Cancer.

[28] Powell HA, Iyen-Omofoman B, Hubbard RB (2013). The association between smoking quantity and lung cancer in men and women. Chest.

[29] Skjefstad K, Grindstad T, Khanehkenari MR (2016). Prognostic relevance of estrogen receptor α, β and aromatase expression in non-small cell lung cancer. Steroids.

[30] Stabile LP, Dacic S, Land SR (2011). Combined analysis of estrogen receptor beta-1 and progesterone receptor expression identifies lung cancer patients with poor outcome. Clin Canc Res.

[31] Labouesse MA, Langhans W, Meyer U (2015). Effects of selective estrogen receptor alpha and beta modulators on prepulse inhibition in male mice. Psychopharmacology (Berl).

[32] Yan M, Rayoo M, Takano EA (2011). Nuclear and cytoplasmic expressions of ERβ1 and ERβ2 are predictive of response to therapy and alters prognosis in familial breast cancers. Breast Cancer Res Treat.

[33] Marquez-Garban DC, Chen HW, Fishbein MC (2007). Estrogen receptor signaling pathways in human non-small cell lung cancer. Steroids.

[34] Nikolos F, Thomas C, Rajapaksa G (2014). ERβ regulates NSCLC phenotypes by controlling oncogenic RAS signaling. Mol Cancer Res.

[35] Pinton G, Thomas W, Bellini P (2010). Estrogen receptor β exerts tumor repressive functions in human malignant pleural mesothelioma via EGFR inactivation and affects response to gefitinib. PLoS One.

[36] Nose N, Sugio K, Oyama T (2009). Association between estrogen receptor β expression and epidermal growth factor receptor mutation in the post-operative prognosis of adenocarcinoma of the lung. J Clin Oncol.

[37] Nose N, Uramoto H, Iwata T (2011). Expression of estrogen receptor beta predicts a clinical response and longer progression-free survival after treatment with EGFR-TKI for adenocarcinoma of the lung. Lung Cancer.

[38] He Q, Zhang M, Zhang J (2015). Correlation between epidermal growth factor receptor mutations and nuclear expression of female hormone receptors in non-small cell lung cancer: a *meta*-analysis. J Thorac Dis.

[39] Wang Z, Li Z, Ding X (2015). ERβ localization influenced outcomes of EGFR-TKI treatment in NSCLC patients with *EGFR* mutations. Sci Rep.

[40] Shen H, Yuan Y, Sun J (2010). Combined tamoxifen and ge?tinib in non-small cell lung cancer shows anti-proliferative effects. Biomed Pharmacother.

[41] Dubey S, Siegfried JM, Traynor AM (2006). Non-small cell lung cancer and breast carcinoma: chemotherapy and beyond. Lancet Oncol.

[42] Liu CM, Chiu KL, Chen TS (2015). Potential therapeutic benefit of combining gefitinib and tamoxifen for treating advanced lung adenocarcinoma. Biomed Res Int.

[43] Garon EB, Pietras RJ, Finn RS (2013). Antiestrogen fulvestrant enhances the antiproliferative effects of epidermal growth factor receptor inhibitors in human non-small cell lung cancer. J Thoracic Oncol.

[b44] 44Garon EB, Siegfried JM, Dubinett SM,* et al*. Results of TORIL-03, a randomized, multicenter phase Ⅱ clinical trial of erlotinib (E) or E + fulvestrant (F) in previously treated advanced non-small cell lung cancer NSCLC. In: Procceding of the 104^th^ Annual Meeting of the American Association for Cancer Research, 2013 Apr 6-10, Washington DC. Philadelphia(PA): AACR. 2013, Abstract 4664.

[45] Siegfried JM, Stabile LP (2014). Estrongenic steroid hormones in lung cancer. Semin Oncol.

[46] Tang H, Liao Y, Chen G (2012). Estrogen upregulates the IGF-1 signaling pathway in lung cancer through estrogen receptor-β. Med Oncol.

[47] Siegfried JM, Farooqui M, Rothenberger NJ (2017). Interaction between the estrogen receptor and fibroblast growth factor receptor pathways in non-small cell lung cancer. Oncotarget.

[48] Che Y, Sun X, Hang C (2014). Influence of tamoxifen or the combnation of tamoxifen and cisplatin on the growth of human lung adenocarcinoma A549 cells. Chinese-German J Clin Oncol.

[49] Hamilton DH, Griner LM, Keller JM (2016). Targeting estrogen receptor signaling with fulvestrant enhances immune and chemotherapy-mediated cytotoxicity of human lung cancer. Clin Cancer Res.

[50] Yu N, Dou L, Li Y (2016). Roles of ERα and ERβ in estrogen-induced DDP chemoresistance in non-small cell lung cancer. Genet Mol Res.

[51] Kohno M, Okamoto T, Suda K (2014). Prognostic and therapeutic implications of aromatase expression in lung adenocarcinomas with EGFR mutations. Clin Cancer Res.

[52] Tanaka K, Shimizu K, Kakegawa S (2016). Prognostic significance of aromatase and estrogen receptor beta expression in EGFR wild-type lung adenocarcinoma. Am J Transl Res.

[53] Mah V, Seligson DB, Li A (2007). Aromatase expression predicts survival in women with early-stage non-small cell lung cancer. Cancer Res.

